# Exploring the Heterogeneity and Trajectories of Positive Functioning Variables, Emotional Distress, and Post-traumatic Growth During Strict Confinement Due to COVID-19

**DOI:** 10.1007/s10902-021-00469-z

**Published:** 2021-11-02

**Authors:** Rosa Mª Baños, Juan J. Garcés, Marta Miragall, Rocío Herrero, Mª Dolores Vara, Emilio Soria-Olivas

**Affiliations:** 1 Department of Personality, Evaluation, and Psychological Treatments, University of Valencia, Valencia, Spain; 2 CIBER Fisiopatología Obesidad Y Nutrición (CIBEROBN), Instituto Carlos III, Madrid, Spain; 3 Instituto Polibienestar, University of Valencia, Valencia, Spain; 4 Intelligent Data Analysis Laboratory (IDAL), Escola Tècnica Superior d’Enginyeria, Universitat de València, Burjassot, Valencia Spain

**Keywords:** COVID-19 pandemic, Clustering analyses, Trajectories, Positive functioning variables, Emotional distress, Post-traumatic growth

## Abstract

COVID-19 pandemic-related confinement may be a fruitful opportunity to use individual resources to deal with it or experience psychological functioning changes. This study aimed to analyze the evolution of different psychological variables during the first coronavirus wave to identify the different psychological response clusters, as well as to keep a follow-up on the changes among these clusters. The sample included 459 Spanish residents (77.8% female, M_age_ = 35.21 years, SD_age_ = 13.00). Participants completed several online self-reported questionnaires to assess positive functioning variables (MLQ, Steger et al. in J Loss Trauma 13(6):511–527, 2006. 10.1080/15325020802173660; GQ-6, McCullough et al. in J Person Soc Psychol 82:112–127, 2002. 10.1037/0022-3514.82.1.112; CD-RISC, Campbell-Sills and Stein in J Traum Stress 20(6):1019–1028, 2007. 10.1002/jts.20271; CLS-H, Chiesi et al. in BMC Psychol 8(1):1–9, 2020. 10.1186/s40359-020-0386-9; SWLS; Diener et al. in J Person Assess, 49(1), 71–75, 1985), emotional distress (PHQ-2, Kroenke et al. in Med Care 41(11):1284–1292, 2003. 10.1097/01.MLR.0000093487.78664.3C; GAD-2, Kroenke et al. in Ann Internal Med 146(5):317–325, 2007. 10.7326/0003-4819-146-5-200703060-00004; PANAS, Watson et al. in J Person Soc Psychol 47:1063–1070, 1988; Perceived Stress, ad hoc), and post-traumatic growth (PTGI-SF; Cann et al. in Anxiety Stress Coping 23(2):127–137, 2010. 10.1080/10615800903094273), four times throughout the 3 months of the confinement. Linear mixed models showed that the scores on positive functioning variables worsened from the beginning of the confinement, while emotional distress and personal strength improved by the end of the state of alarm. Clustering analyses revealed four different patterns of psychological response: “Survival”, “Resurgent”, “Resilient”, and “Thriving” individuals. Four different profiles were identified during mandatory confinement and most participants remained in the same cluster. The “Resilient” cluster gathered the largest number of individuals (30–37%). We conclude that both the heterogeneity of psychological profiles and analysis of positive functioning variables, emotional distress, and post-traumatic growth must be considered to better understand the response to prolonged adverse situations.

**Supplementary Information:**

The online version contains supplementary material available at 10.1007/s10902-021-00469-z.

## Introduction

The COVID-19 pandemic, a worldwide unexpected and unprecedented event, has severely affected the health of communities and welfare systems (Torales et al., 2020). To help prevent the spread of the virus causing COVID-19 (SARS-Cov-2), most governments introduced measures such as quarantine, social/physical distancing, and isolation. In Spain, the first lockdown was imposed on March 14, 2020, and mobility restrictions were implemented until June 21.

This pandemic has led to a global psychological crisis (Duan & Zhu, 2020), generating high levels of stress and/or anxiety in individuals, as a common response to a strange and adverse situation (Roy et al., 2020). Most research on the topic has mainly focused on the adverse psychological effects. For instance, recent meta-analyses report high prevalence of post-traumatic stress disorder (PTSD), anxiety, stress, and depression symptoms among health workers and the general population (Arora et al., 2020; Xiong et al., 2020). Likewise, a cross-sectional study in Spain carried out during the lockdown, has shown high rates of anxiety and depression, affecting almost one third of the general population (Odriozola-González et al., 2020).

Although great research on trauma and significant threat has focused primarily on the negative aspects of mental health (e.g., Neria et al., 2008), only a small percentage of people exposed to the stressor develop a clinically significant psychological disorder (Galea et al., 2003). Recently, Chen and Bonanno (2020) have suggested broadening the research on psychological adjustment to the COVID-19 pandemic by including the “resilience perspective”, instead of only focusing on the psychopathology. In this line, our study focuses on relevant positive aspects of the individual's functioning (e.g., meaning in life, gratitude, compassion, life satisfaction, resilience), as well as post-traumatic growth responses that may be susceptible to change during the confinement due to the COVID-19 pandemic.

Thus, a complementary approach to the psychopathology perspective is to consider the positive aspects of an individual's functioning during potentially stressful events (Tugade & Fredrickson, 2004; Vázquez & Hervás, 2010), and the possibility of growing or improving following the event (Grych et al., 2015; Tamiolaki & Kalaitzaki, 2020). The latter is referred as “Post-Traumatic Growth” (PTG), and involves the positive psychological changes experienced during challenging situations, triggering a shift in thinking and relating to the world, which contribute to a change process (Tedeschi et al., 2004). When PTG occurs, the individual is able to overcome the pre-adversity level of functioning through a process of continuous growth (Ho, 2016). A related concept is “resilience”, defined as an individual characteristic or behavioral resource related to coping mechanisms towards the stressor (McCleary & Figley, 2017) that implies change and adaptation, but not necessarily growth. Being resilient allows adaptation to the stressor and return to pre-adversity normal functioning (Bonanno et al., 2005, 2006). Hence, PTG, unlike resilience, is an adaptive response, involving growth and gain from the traumatic event.

Other positive functioning variables have also been shown to be key resources when dealing with adversity, such as the COVID-19 pandemic. In this line, a “meaningful life” has been shown to allow individuals to re-evaluate traumatic events positively, empower the psychological resources needed to rediscover themselves, restore their essential assumptive world, and orient themselves towards future goals (Updegraff et al., 2008). Moreover, “gratitude” leads to spiritual deepening and a greater sense of one's life value following trauma (Vernon et al., 2009). Besides, “compassion” toward others enables healing and recognizing their own personal strength in the face of adversity (Malhotra & Chebiyan, 2016). Furthermore, “life satisfaction” has been found to serve as a buffer against emotional distress regarding unpredictable threats (Trzebiński et al., 2020).

As stated above, positive responses can be built up with time to confront an adverse situation. In addition, theoretical approaches to trauma point out the relevance of assessing the different reaction patterns or trajectories in adverse situations for extended periods (Bonanno & Mancini, 2012). Diverse response patterns have been shown when facing adverse situations (Bonanno, 2004; Bonanno et al., 2008; Hobfoll et al., 2009; Lau et al., 2006; Lepore & Revenson, 2006; Smith & Ehlers, 2020). Galatzer-Levy et al. (2018) carried out a review of several studies aimed at analyzing the nature and prevalence of common trajectories of response to major life stressors. Prototypical trajectories of resilience, chronic dysfunction, delayed reactions, and recovery were found across different contextual factors, with resilience (i.e., stable psychological and physical health over the course of the adverse event) being the most common response. So far, little is known about the psychological profiles that arose during the COVID-19 pandemic when considering the interaction of emotional distress, positive functioning variables, PTG, and emotional distress. Moreover, it could be also very useful to analyze how these psychological profiles changed throughout confinement, examining the trajectories over time and not only for cross-sectional periods.

To date, limited research has been done on assessing the changes over time regarding psychological functioning and specifically on adaptive psychological responses during the lockdown period during the first coronavirus wave (March-June, 2020). Identifying the different psychological response profiles to this adverse event (strict lockdown) and the changes across the evolution of the pandemic over time, may allow to better understand the responses from individuals towards adverse situations, and refining the interventions to improve mental wellbeing, promote PTG, and reduce the risk of psychopathology in future waves or similar crises. This study was aimed to assess several positive variables (meaning in life, gratitude, resilience, compassion towards strangers, and life satisfaction) and PTG (new possibilities in life, closer relationships to others, increases in personal strength, appreciation of life, and spiritual changes), as well as emotional distress (perceived stress, symptoms of anxiety and depression, and positive and negative affects) in a three-month longitudinal study throughout the entire enforced and mandatory confinement due to the COVID-19 pandemic in Spain.^1^ Specifically, the objectives of this study are: (1) to analyze the evolution of different psychological variables during the lockdown; (2) to identify if there are different psychological responses (or clusters) towards the COVID-19 pandemic-related confinement and describe them based on positive functioning variables, emotional distress, and PTG; and (3) to identify the changes between clusters throughout the evolution of the lockdown period (three months) among the Spanish population. Regarding these objectives, we hypothesize that: (1) Given the challenging and chronic stressor of the confinement -and considering both the “psychopathology” (Odriozola-González et al., 2020) and “resilience” (Chen & Bonanno, 2020) perspectives-, we expect that positive functioning variables will decrease, emotional distress variables will increase, and PTG will increase across the confinement; (2) given individual differences, strict confinement will impact differently on people, and therefore, different profiles (i.e., clusters) will be identified^2^; and (3) given the different trajectories found in previous studies when dealing with stressors (Galatzer-Levy et al., 2018), and the length and unexpected changes in restrictions during the confinement, a static evolution in the participants is not expected (i.e., we expect that the percentage of participants in each cluster will change over time).

## Method

### Participants

The sample consisted of 493 volunteers. Inclusion criteria were: (1) age ≥ 18 years and (2) be living in Spain at the time of confinement. No exclusion criteria were established. Each participant answered the survey four times. Sample size decreased over time due to dropouts. Period 1 included 493 participants (78.1% female, *M*_*age*_ = 35.40, *SD*_*age*_ = 13.06); Period 2, 231 participants (81.8% female, M_age_ = 35.95, *SD*_*age*_ = 13.18); Period 3, 206 participants (83.0% female, *M*_*age*_ = 37.15, *SD*_*age*_ = 13.58); and Period 4, 184 participants (83.2% female, *M*_*age*_ = 36.48, *SD*_*age*_ = 13.40). The final sample analyzed is described in Table 1.^3^

**Table 1:** Demographic characteristics of the sample

	Period 1 N = 459	Period 2 N = 221	Period 3 N = 191	Period 4 N = 162
Sex (%women)	77.8%	82.4%	83.2%	82.7%
Age (years) *M (SD)*	35.21 (13.00)	35.90 (13.01)	37.28 (13.45)	36.78 (16.46)
18–24 years old	25.5%	20.8%	19.9%	20.4%
25–35 years old	33.8%	37.1%	34.0%	35.2%
36–50 years old	24.4%	25.8%	26.2%	25.9%
> 50 years old	16.3%	16.3%	19.9%	18.5%
Diagnosis of mental illness (% yes)	6.5%	7.2%	6.8%	4.3%
Diagnosis of chronic disease (% yes)	18.5%	21.3%	21.5%	24.1%
Marital status				
Single	27.7%	27.6%	24.1%	27.2%
In a relationship	37.3%	36.7%	39.8%	36.4%
Married	26.1%	23.5%	24.6%	24.7%
Divorced/separated	7.0%	9.5%	8.4%	9.3%
Widowed	0.7%	1.4%	1.6%	1.9%
Other	1.3%	1.4%	1.6%	0.6%
Monetary income				
Below the mean	37.0%	37.6%	35.1%	34.6%
At the mean	50.1%	49.8%	52.9%	53.1%
Above the mean	12.9%	12.7%	12.0%	12.3%
Employment situation				
Employee (permanent job)	37.3%	34.4%	35.1%	38.9%
Employee (temporal job)	17.4%	19.0%	16.8%	15.4%
Freelancer	5.0%	3.6%	4.2%	3.7%
Job seeker	8.9%	8.6%	7.3%	6.2%
Student	23.1%	23.1%	22.0%	21.6%
Other	8.3%	11.3%	14.7%	14.2%
Healthcare professional				
Yes (working currently)	8.3%	8.6%	8.4%	8.0%
Yes (but not working currently)	12.2%	14.5%	15.2%	12.3%
Employment situation during coronavirus crisis				
Teleworking	35.3%	33.0%	33.0%	35.8%
Regular workplace (partial time)	3.5%	4.5%	4.7%	4.3%
Regular workplace (full time)	8.5%	7.7%	7.9%	6.8%
Studying	25.7%	24.9%	23.6%	23.5%
Unemployed	27.0%	29.9%	30.9%	29.6%

### Procedure

Participants were invited to participate in the study to evaluate potential positive psychological factors associated with the confinement due to the COVID-19 pandemic, in Spain. The web-based tool Qualtrics was used to complete the surveys. The information was published on social networks (e.g., WhatsApp, Instagram). A raffle was held to encourage participation. After signing the informed consent, volunteers were asked to complete the surveys four times, which included several questionnaires.

**Fig. 1:**
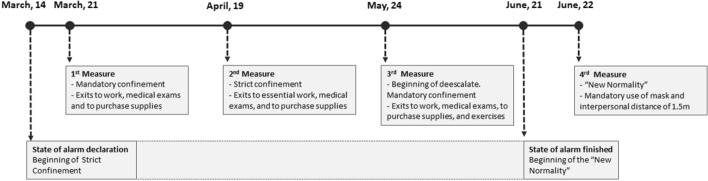
State of alarm milestones in Spain

The survey for Period 1 was completed between March 2 and April 15 (when the strictest measures were implemented; https://www.boe.es/eli/es/rd/2020/03/14/463/con), and three more times after that (more less every month), based on the occurrence of significant milestones and until the state of alarm was over in Spain. Period 2 was on April 19 (when the strictest measures ended, but confinement was still mandatory), Period 3 was on May 24 (at the beginning of de-escalation; https://www.boe.es/eli/es/o/2020/04/30/snd380), and Period 4 was after June 22 (once the “new normality” started; https://www.boe.es/eli/es/rdl/2020/06/09/21) (see Fig. 1).

The study was conducted in accordance with the Declaration of Helsinki (World Medical Association, 2013) and approved by the ethical committee of the University of Valencia (Spain) (register number: 1593681212393).

### Measures

#### Socio-demographic Characteristics

Participants included information regarding their age, sex, marital status, monetary incomes, diagnosis of mental and chronic illness, occupational situation, employment situation, and if they were working as healthcare professionals.

#### Positive Functioning Measures

##### Meaning in Life

The Meaning in Life Questionnaire (MLQ: Steger et al., 2006, 2008) uses 10 items aimed at assessing two dimensions in life: (1) presence of meaning (MLQ-P) and (2) search for meaning (MLQ-S). Each item is rated on a seven-point Likert scale. The internal consistencies for MLQ-P ranged between α = 0.90 and α = 0.91, and for MLQ-S between α = 0.93 and α = 0.96 over time.

##### Gratitude

The Gratitude Questionnaire-6 (GQ-6; McCullough et al., 2002; Magallares et al., 2018) contains six items that allow assessing the proneness to experience gratitude in daily life. Items are rated on a seven-point Likert scale. The internal consistencies ranged between α = 0.71 and α = 0.83 over time.

##### Resilience

The Connor-Davidson Resilience Scale (CD-RISC; Campbell-Sills & Stein, 2007; Notario-Pacheco et al., 2011) contains 10 items that measure resilience. Items are rated on a five-point Likert-type scale. The internal consistencies ranged between α = 0.87 and α = 0.89 over time.

##### Compassion

The Compassionate Love Scale for Humanity (CLS-H; Chiesi et al., 2020; Spanish translation by the authors) contains nine items that assess the degree to which an individual feels compassion or altruistic love towards strangers, selfless caring, and the motivation to help humanity. Items are rated on a six-point Likert scale. The internal consistencies ranged between α = 0.92 and α = 0.95 over time.

##### Life Satisfaction

The Satisfaction with Life Scale (SWLS; Diener et al., 1985; Vázquez et al., 2013) contains five items that assess the global cognitive component of subjective well-being. Items are rated on a seven-point Likert scale. The internal consistencies ranged between α = 0.86 and α = 0.91 over time.

#### Emotional Distress Measures

##### Depressive Symptoms

The Patient Health Questionnaire-2 (PHQ-2; Kroenke et al., 2003; Rodríguez-Muñoz et al., 2017) contains two items that assess the symptoms of depression. Items are scored on a four-point Likert scale. Reliability ranged between *r*_*SB* =_ 0.61 and *r*_*SB* =_ 0.68 over time.^4^

##### Anxiety Symptoms

The Generalized Anxiety Disorder Questionnaire-2 (GAD-2; Kroenke et al., 2007; García-Campayo et al., 2012) contains two items that assess the symptoms of anxiety. Items are scored on a four-point Likert scale. Reliability ranged between *r*_*SB* =_ 0.52 and *r*_*SB* =_ 0.68 over time.

##### Positive and Negative Affect

Positive and Negative Affect Schedule (PANAS; López-Gómez et al., 2015; Watson et al., 1988) contains 20 items that assess two independent dimensions: positive affect (PANAS positive) and negative affect (PANAS negative). Items are scored on a five-point Likert scale. The internal consistencies for PANAS positive ranged between α = 0.92 and α = 0.95 and for PANAS negative ranged between α = 0.88 and α = 0.92 over time.

##### Perceived Stress

An ad hoc visual analog scale with two items was developed to evaluate the level in which current life is perceived as stressful (“I have felt that I can deal with all the things I should do”; “I have managed the small daily problems”). Item responses are rated from 1 (never) to 5 (very often). Reliability ranged between *r*_*SB* =_ 0.57 and *r*_*SB* =_ 0.67 over time.

#### Post-traumatic Growth Measure

##### Post-traumatic Growth

The short form of the Post-traumatic Growth Inventory (PTGI-SF; Cann et al., 2010; Cárdenas et al., 2015) contains 10 items that measure the extent to which individuals report positive life changes in the aftermath of a major life crisis. Five dimensions of PTGI are assessed with two items: new possibilities, relating to others, personal strength, appreciation of life, and spiritual change. Items are rated on a six-point Likert scale. The reliability for “new possibilities”, “relating to others”, “personal strength”, “appreciation of life” and “spiritual change” ranged between *r*_*SB* =_ 0.60 to *r*_*SB* =_ 0.66, *r*_*SB* =_ 0.65 to *r*_*SB* =_ 0.80, *r*_*SB* =_ 0.70 to *r*_*SB* =_ 0.81, *r*_*SB* =_ 0.72 to *r*_*SB* =_ 0.79, and *r*_*SB* =_ 0.40 to *r*_*SB* =_ 0.57 over time, respectively.

### Data Analyses

Statistical analyses were performed using the SPSS software v.26 and the R 3.6.3 program. The analyses were performed with the “completers”.^5^

First, descriptive statistics were calculated to explore the demographic characteristics of the sample in the four assessment periods. Second, linear mixed models were employed for each study variable using the MIXED procedure with one random intercept per subject and without ad hoc imputation. An identity covariance structure was specified to model the covariance structure of the random intercept. For each outcome, time was treated as a within-group factor. Pairwise comparisons were followed by adjustments using the Bonferroni correction for multiple comparisons.^6^

Third, a clustering analysis was carried out. Clustering—also termed segmentation of data—belongs to the set of techniques known in machine learning as unsupervised learning (Hastie et al., 2017); it is aimed at precisely dividing a set of samples into several groups or clusters, based on the patterns within the data itself. The K-means algorithm was used to perform the clustering analyses, a non-model-based method that uses optimization algorithms to define participants' clusters (Forgy, 1965). K-means was chosen, as is a widely used clustering technique that has been extensively used in the field of Psychology (e.g., Clatworthy et al., 2005; Grant et al., 2020; Zakharov, 2016).

In K-means, each point (i.e., each participant of the sample) is assigned to the cluster whose center (also called centroid) is nearest. The centroid of the cluster is the sum of the deviation of each variable compared with the centroid values and K is the number of clusters. K-means does not make use of any mathematical transformation to arrive at the clusters and the conclusions are drawn directly from the values of the variables in the dataset. To determine the optimal number of groups (K), the elbow technique (“elbow plot” or “elbow curve”) was used (Hartigan & Wong, 1979), which calculates the sum of the quadratic distances of each sample to the centroid of its cluster for different K. The elbow technique generates a graph, in which the different used K's are represented on the x-axis and the calculated value on the y-axis. The criterion states that the optimal number of clusters is the one in which adding another cluster does not add significant information (i.e., the inflection point or elbow where the variation in the sum begins to be very small indicates a good K value).

General K-means procedure is as follows: (1) the position of the centroids is set up randomly, and samples are assigned to each cluster according to the closest distance metric; and (2) an iterative process is established in which the centroids are recalculated, and the samples reassigned until one or several stop criteria are met (i.e., not variations in cluster assignment from the previous iteration, or to reach a maximum of 10 iterations). In this study, 16 variables (described in 2.3: MLQ-P, MLQ-S, GQ-6, CD-RISC, CLS-H, SWLS, PHQ-2, GAD-2, PANAS positive, PANAS negative, Perceived Stress, and the five sub-dimensions of PTGI-SF) were used to set up the groupings. Groupings were made for four study assessments. For Period 1, the centroids were randomly chosen. We used a code seed to ensure the cluster reproducibility. The centroids obtained in Period 1 were provided as the starting point for Period 2 clustering process, Period 2 centroids for Period 3, and so on. By this procedure, we preserved cluster coherence between periods, while at the same time, the particularities of each period updated the centroids and the participants allocated in each cluster through the iterative algorithm. Thus, the four clusters were recalculated and the sample distributed in the different clusters for each period. To facilitate the interpretation of the clusters, we calculated the direct scores (mean and standard deviation) and the standard scores ranging from 0 to 1. The standard scores were calculated considering the minimum and maximum score of each measurement scales and the following formula Z_i_ = $\frac{Xi-min(x) }{max\left(x\right) -min(x)}$. Zi values were categorized as “low”, “medium–low”, “medium”, “medium–high”, and “high” for each questionnaire (see Supplemental information 1).

Finally, to analyze the age and sex in the different clusters at each period, unifactorial ANOVAs (for continuous variables) and chi-squared tests (for categorical variables) were calculated. Moreover, to analyze the differences in the number of changes over time depending on sociodemographic variables and the cluster in which participants started at Period 1, several unifactorial ANOVAs and chi-squared tests were calculated. The percentage of participants who migrated among clusters and the specific trajectories that followed participants over time were determined.

## Results

### Descriptive Statistics of the Sample

The demographic characteristics of the sample for each measurement period are shown in Table 1.

### Changes in Positive Functioning Variables, Emotional Distress, and Post-traumatic Growth (PTG) over Time

**Fig. 2:**
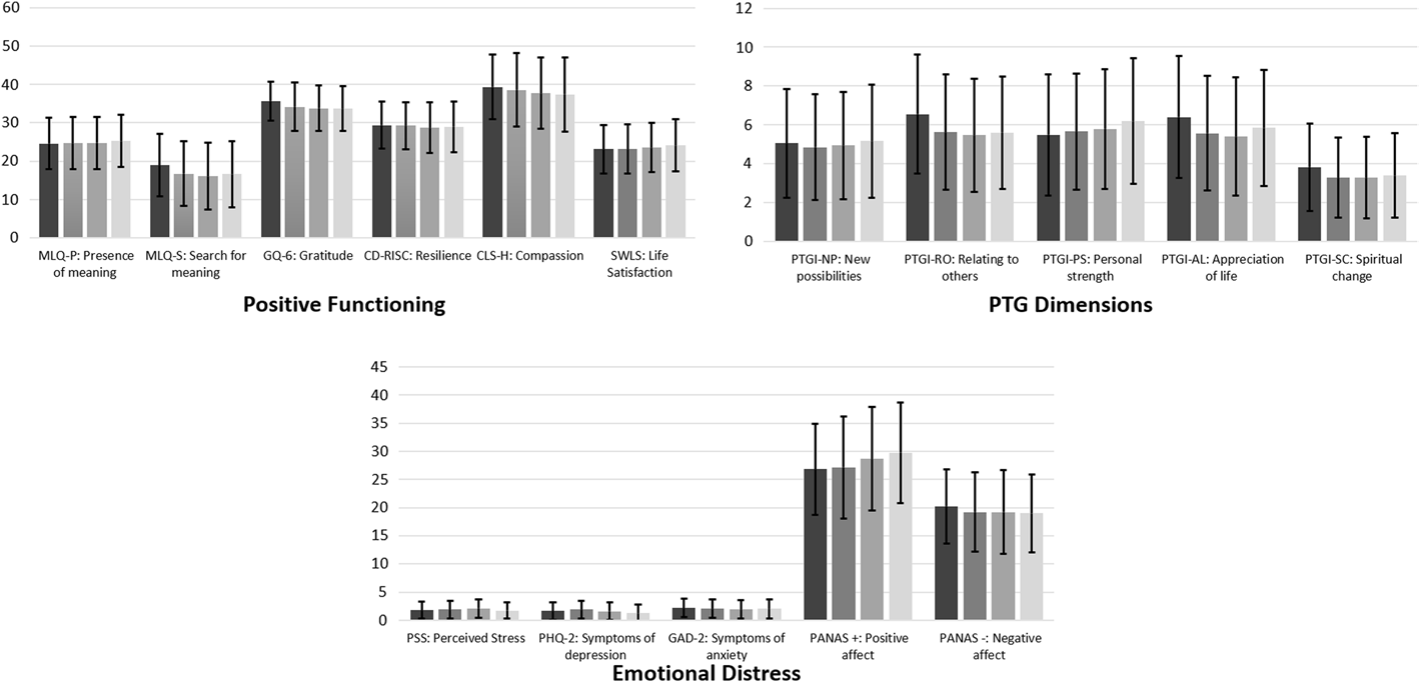
Differences in study variables over time. *Notes*: Error bars represent ± 1 standard deviation. Colors represent the periods: MLQ= The Meaning in Life Questionnaire; GQ-6=The Gratitude Questionnaire-6; CD-RISC= The Connor-Davidson Resilience Scale; CLS-H= The Compassionate Love Scale for Humanity; SWLS= The Satisfaction with Life Scale; PS= Perceived Stress; PHQ-2= The Patient Health Questionnaire-2; GAD – 2= The Generalized Anxiety Disorder Questionnaire-2; PANAS Positive and Negative Affect Schedule; PTGI-SF= short form of the Posttraumatic Growth Inventory

Main effects of time were found on several positive functioning variables, emotional distress, and PTG. Table 2 and Fig. 2 show the descriptive statistics of each assessed period, the results of the linear mixed models, and the Bonferroni post hoc comparisons.

**Table 2:** Differences in the study variables over time

		Period 1 *M (SD)*	Period 2 *M (SD)*	Period 3 *M (SD)*	Period 4 *M (SD)*	Linear mixed model	Bonferroni post-hoc comparison
Positive functioning variables	1. MLQ-P: Presence of meaning	24.56 (6.69)	24.70 (6.72)	24.73 (6.80)	25.26 (6.81)	*F*(3, 616.01) = 1.76, *p* = .153	−
	2. MLQ-S: Search for meaning	18.95 (8.10)	16.72 (8.37)	16.02 (8.75)	16.62 (8.65)	*F*(3, 633.58) = 15.68, *p* < .001	T1 > T2 & T3 & T4
	3. GQ-6: Gratitude	35.65 (5.08)	34.17 (6.25)	33.78 (5.89)	33.68 (5.88)	*F*(3, 652.67) = 29.03, *p* < .001	T1 > T2 & T3 & T4
	4. CD-RISC: Resilience	29.39 (6.20)	29.29 (6.13)	28.70 (6.56)	29.01 (6.61)	*F*(3, 611.34) = 4.41, *p* = .004	T1 > T2 & T3
	5. CLS-H: Compassion	39.34 (8.50)	38.57 (9.54)	37.68 (9.28)	37.38 (9.73)	*F*(3, 614.12) = 9.70, *p* < .001	T1 > T2 & T3 & T4
	6. SWLS: Life Satisfaction	23.08 (6.39)	23.15 (6.41)	23.55 (6.41)	24.08 (6.83)	*F*(3, 605.66) = 2.11, *p* = .098	−
Emotional distress	7. PSS: Perceived Stress	1.77 (1.49)	1.88 (1.53)	2.07 (1.63)	1.72 (1.48)	*F*(3, 691.63) = 5.67, *p* < .001	T1 < T3; T3 > T4
	8. PHQ-2: Symptoms of depression	1.63 (1.59)	1.89 (1.53)	1.58 (1.59)	1.25 (1.52)	*F*(3, 688.55) = 13.66, *p* < .001	T1 < T2; T2 > T3 & T4 T1 & T3 > T4
	9. GAD-2: Symptoms of anxiety	2.19 (1.68)	2.02 (1.65)	1.92 (1.60)	2.02 (1.67)	*F*(3, 688.12) = 1.08, *p* = .358	−
	10. PANAS + : Positive affect	26.82 (8.08)	27.14 (9.02)	28.67 (9.20)	29.76 (8.99)	*F*(3, 678.78) = 12.91, *p* < .001	T1 & T2 & T3 < T4
	11. PANAS -: Negative affect	20.25 (6.60)	19.21 (7.06)	19.23 (7.42)	19.01 (6.93)	*F*(3, 682.30) = 3.04, *p* = .029	T1 > T4
PTG dimensions	12. PTGI-NP: New possibilities	5.05 (2.80)	4.84 (2.72)	4.93 (2.75)	5.16 (2.91)	*F*(3, 640.48) = 1.01, *p* = .388	−
	13. PTGI-RO: Relating to others	6.54 (3.06)	5.62 (2.98)	5.46 (2.91)	5.59 (2.88)	*F*(3, 634.74) = 17.61, *p* < .001	T1 > T2 & T3 & T4
	14. PTGI-PS: Personal strength	5.48 (3.12)	5.65 (2.99)	5.78 (3.09)	6.19 (3.22)	*F*(3, 632.84) = 6.21, *p* < .001	T1 & T2 < T4
	15. PTGI-AL: Appreciation of life	6.40 (3.13)	5.56 (2.95)	5.40 (3.06)	5.84 (2.99)	*F*(3, 635.05) = 8.35, *p* < .001	T1 > T2 & T3
	16. PTGI-SC: Spiritual change	3.82 (2.24)	3.28 (2.06)	3.28 (2.11)	3.39 (2.17)	*F*(3, 614.73) = 4.02, *p* = .008	T1 > T2 & T3

MLQ = The Meaning in Life Questionnaire; GQ-6 = The Gratitude Questionnaire-6; CD-RISC = The Connor-Davidson Resilience Scale; CLS-H = The Compassionate Love Scale for Humanity; SWLS = The Satisfaction with Life Scale; PS = Perceived Stress; PHQ-2 = The Patient Health Questionnaire-2; GAD – 2 = The Generalized Anxiety Disorder Questionnaire-2; PANAS Positive and Negative Affect Schedule; PTGI-SF = short form of the Posttraumatic Growth Inventory

Regarding positive functioning variables, scores were higher in Period 1 in comparison to Periods 2–4 for search of meaning, gratitude, resilience, and compassion towards strangers. The scores on presence of meaning or life satisfaction were found to remain stable over time.

Regarding emotional distress, results showed that scores were higher in Period 1 in comparison to Period 4 on negative affect, while positive affect was significantly higher in Period 4 in comparison to Periods 1–3. Symptoms of depression increased significantly in Period 2 in comparison to Periods 1, 3 and 4, but depression scores decreased significantly in Period 4 in comparison to Periods 1 and 3. Perceived stress significantly increased in Period 3 in comparison to period 1, although in Period 4, the perceived stress significantly decreased in comparison to period 3, without differences with Period 1. Symptoms of anxiety remained stable over time.

Regarding PTG, scores on dimensions “relating to others”, “appreciation of life”, and “spiritual change”, were higher in Period 1 in comparison to Periods 2–4. However, personal strength was significantly higher in Period 4 in comparison to periods 1–2. The scores on “new possibilities” remained stable over time.

### Clustering Analyses: Psychological Profiles over Time

A cluster analysis with the measures obtained for Period 1 (N = 459) was performed following the k-means algorithm (Hartigan et al., 1979). A solution with four distinct clusters (“Survival”, “Resurgent”, “Resilient”, and “Thriving”) was chosen (see Supplementary Information 2). The solution with four clusters was calculated for each of the four period, and the resulting clusters were relatively stable over the assessed periods.

**Fig. 3:**
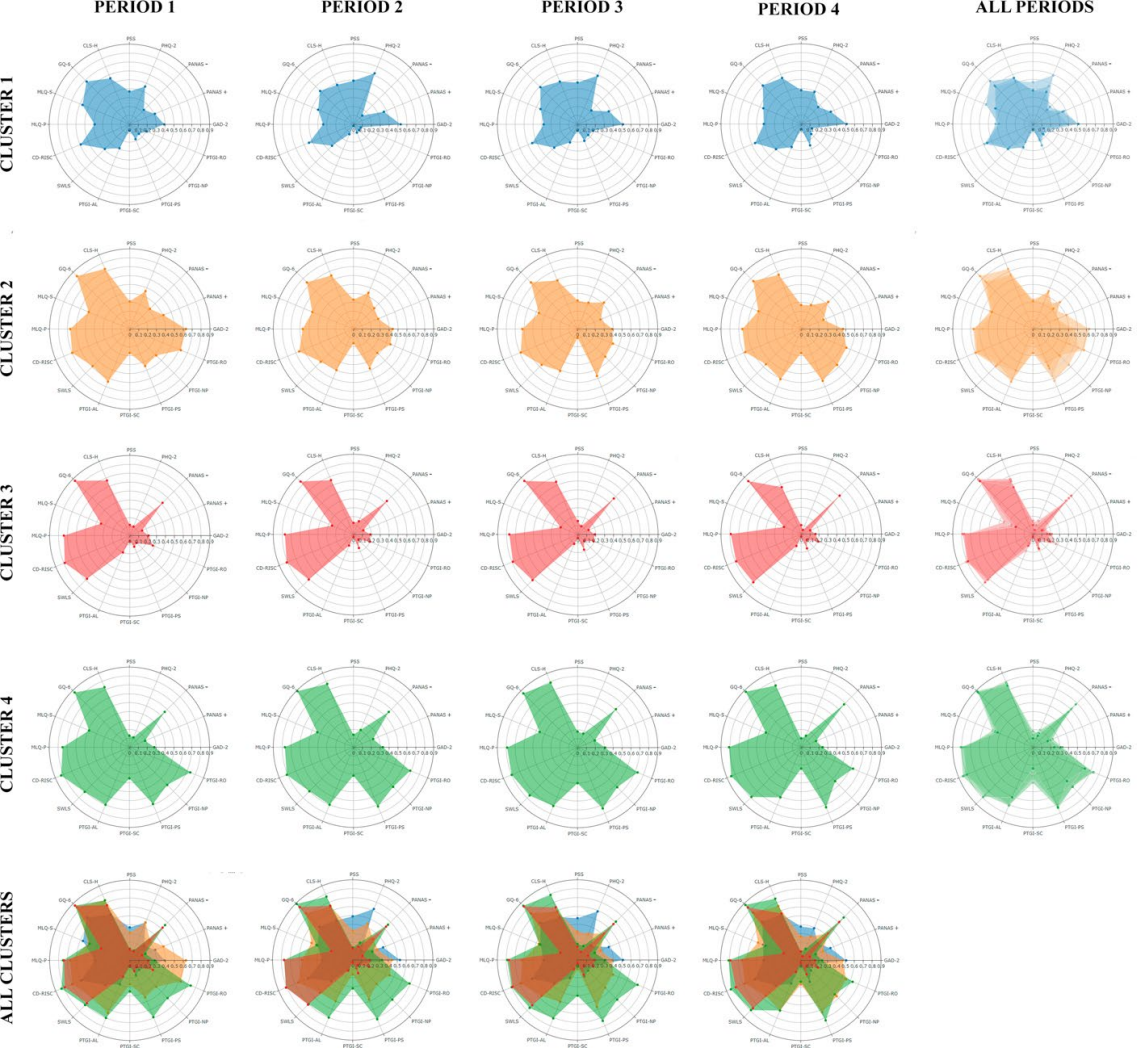
Graphical representation of the standardized scores (0–1) for each cluster and assessed periods. *Notes:* MLQ = The Meaning in Life Questionnaire; GQ-6 = The Gratitude Questionnaire-6; CD-RISC = The Connor-Davidson Resilience Scale; CLS-H = The Compassionate Love Scale for Humanity; SWLS = The Satisfaction with Life Scale; PSS-2 = The Perceived Stress Scale; PHQ-2 = The Patient Health Questionnaire-2; GAD- 2 = The Generalized Anxiety Disorder Questionnaire-2; PANAS Positive and Negative Affect Schedule; PTGI-SF = short form of the Post-traumatic Growth Inventory; NP = New possibilities; RO = Relating to others; PS = Personal strength; AL = Appreciation of life; SC = Spiritual change

Below we present a descriptive analysis of the clusters using standardized scores ranging from 0 to 1. Direct and standard scores (0–1) of each cluster are illustrated in Table 3 and Fig. 3.

**Table 3:**
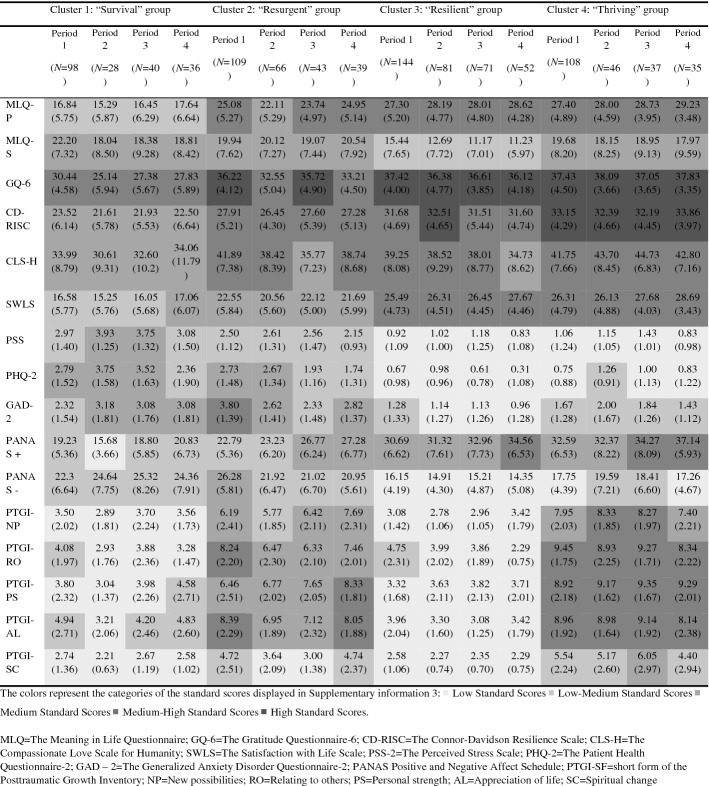
Direct scores of each cluster in each study variable

***Cluster 1:*** At the beginning of the lockdown (Period 1), the scores of this group ranged from “low-medium” to “medium–high” on positive functioning variables and emotional distress, while the scores on PTG ranged from “low” to “low-medium”. Overall, the scores were quite stable during the three months. A slight increase in emotional distress in Periods 2 and 3 was observed, and even a bit higher in depression and positive affect in Period 2, returning to the initial level (Period 1) once the lockdown period ended (Period 4). The increase in PTG remained “low” and “low-medium” at all periods. This cluster was labeled “***Survival***” because individuals showed no high emotional distress nor presented high positive functioning, and although their scores got slightly worse when the confinement was stricter, they returned to their initial state after lockdown was over. Furthermore, they did not show an increase in PTG during and after lockdown.

***Cluster 2:*** At the beginning of the lockdown (Period 1), the scores of this group ranged from “medium” to “high” on positive functioning variables, remaining stable throughout all the periods. The higher scores were observed for gratitude. Regarding emotional distress, the scores ranged from “low-medium” to “medium–high”. However, over the following two months (Periods 2 and 3), emotional distress scores related to depression, anxiety, and negative effects slightly decreased, while perceived stress remained stable and positive affects slightly increased. Finally, for PTG, scores ranged from “medium” to “medium–high”. Slight variations in PTG scores were also observed in Periods 2 and 3 (e.g., decreases in new possibilities, relating to others, spiritual change, and appreciation of life scores). Nevertheless, these scores ranged from “low-medium” to “medium–high” (except for the scores in spirituality that decreased to “low”). Once the lockdown was over (Period 4), slight increases with respect to Periods 2 and 3 were observed in personal strength, appreciation of life, and spiritual change. This cluster was labeled “***Resurgent***” because individuals showed moderate scores in PTG (i.e., the second cluster that experienced it the most) despite not having too high scores in positive functioning variables (only moderate) and experiencing moderate emotional distress.

***Cluster 3:*** At the beginning of the lockdown (Period 1), the scores of this group ranged from “medium–high” to “high” on positive functioning variables. The higher scores were for gratitude, and in Period 2, for resilience. Moreover, this group showed “low” scores on emotional distress (except for anxiety that was “low-medium”), as well as “medium” scores on positive affect. Similarly, “low” scores were obtained in PTG (except for relating to others with “low-medium” scores). These scores remained stable throughout and after the lockdown (Periods 2–4). This cluster was labeled “***Resilient***” because individuals showed high positive functioning, with low emotional distress throughout the pandemic, but nevertheless, low PTG.

***Cluster 4:*** At the beginning of the lockdown (Period 1), the scores of this group ranged from “medium–high” to “high” on positive functioning variables. The higher scores were for gratitude and resilience. As for emotional distress, they had “low” or “low-medium” scores, and “medium” scores in positive affect. Overall, the scores for these variables remained stable throughout and after the lockdown (Periods 2–4). Although depression and negative affect slighted increased in Period 2, these scores (and positive affect) improved in Periods 3 and 4, even in comparison to Period 1. Finally, this group showed “low-medium” and “medium–high” scores on PTG. The scores relating to others, personal strength, and appreciation of life remained “medium–high” over the whole period. This cluster was labeled “***Thriving***” because individuals showed high positive functioning (with a slight increase in positive affect during confinement), with moderate emotional distress. This group showed the greatest PTG during the entire confinement.

### Cluster Differences Based on Age and Sex

Regarding age, there were significant differences among clusters for Period 1, *F*(3, 455) = 15.85, *p* < 0.001, *η*_*p*_^2^ = 0.10; Period 2, *F*(3, 217) = 12.42, *p* < 0.001, *η*_*p*_^2^ = 0.15; Period 3, *F*(3, 187) = 7.47, *p* < 0.001, *η*_*p*_^2^ = 0.11; and Period 4, *F*(3, 158) = 9.27, *p* < 0.001, *η*_*p*_^2^ = 0.15. Post hoc comparison using the Bonferroni correction showed that: (1) for Period 1, mean age of the “Thriving” (*M* = 38.04, *SD* = 12.49) and “Resilient” (*M* = 39.29, *SD* = 14.29) groups were higher than for the “Survival” (*M* = 29.83, *SD* = 10.05) (*p* < 0.001 and *p* < 0.001, respectively) and “Resurgent” (*M* = 31.87, *SD* = 11.54) (*p* = 0.002 and *p* < 0.001, respectively) groups; (2) for Period 2, mean age of the “Resilient” group (*M* = 42.17, *SD* = 13.72) was higher than the “Survival” (*M* = 29.18, *SD* = 9.89; *p* < 0.001), “Resurgent” (*M* = 32.29, *SD* = 11.63; *p* < 0.001), and “Thriving” (*M* = 34.15, *SD* = 10.84; *p* = 0.002) groups; (3) for Period 3, mean age of the “Resilient” group (*M* = 42.52, *SD* = 14.48) was higher than for the “Survival” (*M* = 31.30, *SD* = 10.87; *p* < 0.001), “Resurgent” (*M* = 34.72, *SD* = 11.70; *p* = 0.011) groups; and (4) for Period 4, mean age of the “Resilient” group (*M* = 44.27, *SD* = 14.45; *p* < 0.001) was higher than for the “Survival” (*M* = 32.19, *SD* = 11.83; *p* < 0.001), “Resurgent” (*M* = 33.36, *SD* = 11.32; *p* < 0.001), and “Thriving” (*M* = 34.20, *SD* = 11.36; *p* = 0.002) groups.

Concerning sex, there were no significant inter-cluster differences for Period 1, χ^2^ (3, *N* = 454) = 5.39, *p* = 0.150. However, there were significant sex-related differences between clusters for Period 2, χ^2^(3, *N* = 220) = 15.38, *p* = 0.002; for Period 3, χ^2^(3, *N* = 190) = 7.92, *p* = 0.047; and (marginally significant) for Period 4, χ^2^(3, *N* = 162) = 7.39, *p* = 0.058. For periods 2–4, there was a greater percentage of men than women in the “Resilient” group (*Period 2:* 60.5% vs. 31.9%, Adjusted Standardized Residuals = 3.3; *Period 3:* 58.1% vs. 33.3%, Adjusted Standardized Residuals = 2.6; *Period 4:* 53.6% vs. 27.6%, Adjusted Standardized Residuals = 2.7), as well as lower percentage of men than women in the “Resurgent” group for Period 2 (7.9% vs. 34.6%, Adjusted Standardized Residuals = 3.3).

### Follow-Up of Changes Between Clusters over Time

**Fig. 4:**
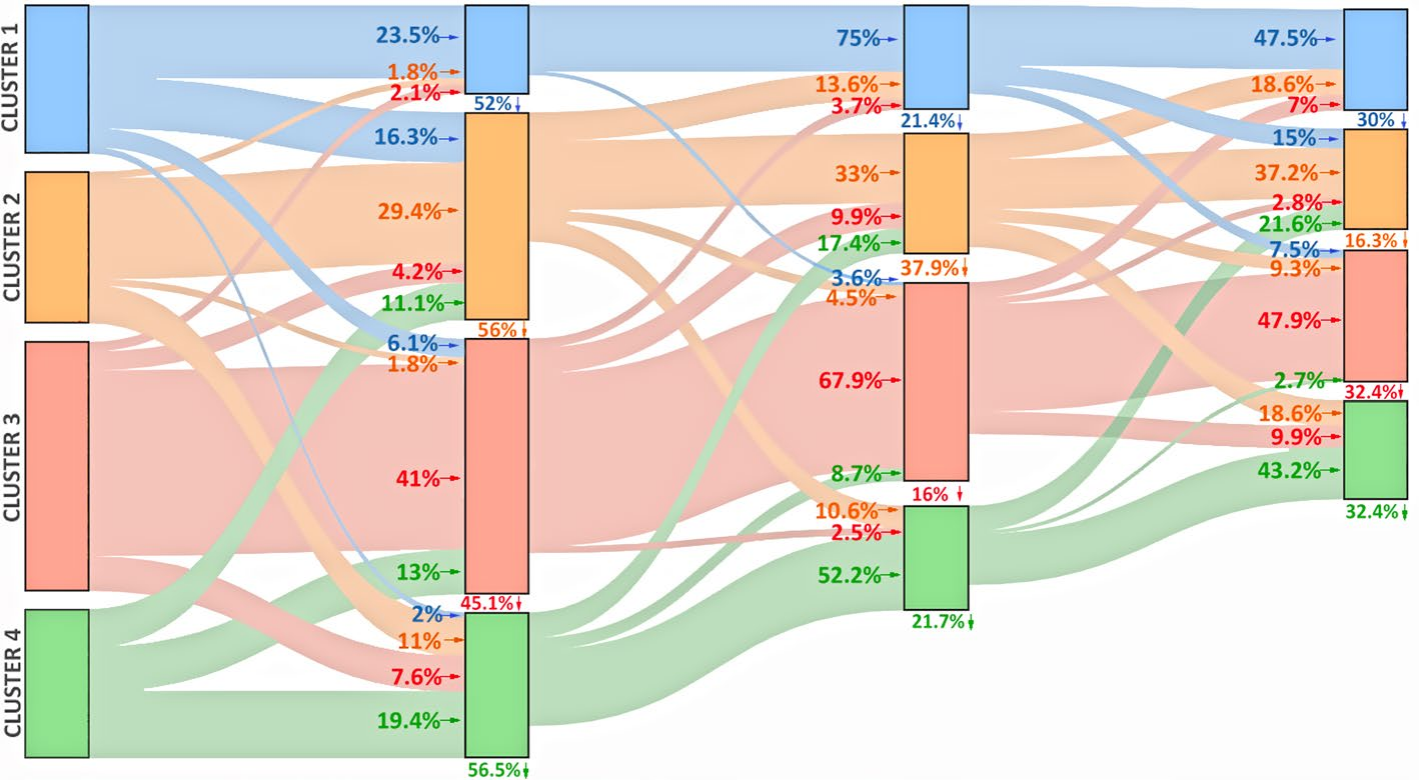
Graphical representation of the percentage of participants that migrated among clusters over time. *Notes:* Cluster 1 = “Survival” group; Cluster 2 = “Resurgent” group; Cluster 3 = “Resilient” group; Cluster 4 = “Thriving” group. The first column represents Period 1, the second column represents Period 2, the third column represents Period 3, and the fourth column represents Period 4

The graphical representation of the percentage of participants that migrated among clusters over time is shown in Fig. 4. The “Survival” (Cluster 1), “Resurgent” (Cluster 2), and “Thriving” (Cluster 4) groups had similar numbers of participants for Period 1 (around 20% in each group). The “Resilient” group (Cluster 3) had the largest number of participants at the beginning (around 30%) and over time (around 31–37%).

The highest percentage of dropouts occurred in Period 2, around 50% of the participants of each cluster did not continue the study. The graphical representation and percentage of participants that migrated among clusters throughout Periods 1–4 are illustrated in Fig. 3 and Supplementary information 3.

The trajectories of the “Survival” (Cluster 1) and “Thriving” (Cluster 4) groups were very similar. Approximately, half of the participants (23.5% for Cluster 1 and 19.4% for Cluster 4) in these groups remained in the same cluster during Period 2, while the other half of the participants (24.4% for Cluster 1 and 24.1% for Cluster 3) migrated to other clusters. Most participants from Cluster 1 migrated to Cluster 2 (“Resurgent” 16.3%), while most participants in Cluster 4 migrated to Cluster 2 (“Resurgent” 11.1%) and Cluster 3 (“Resilients”13.0%). Later, Clusters 1 and 4 remained stable over time (75.0% and 47.5% remained in Cluster 1, and 52.2% and 43.2% remained in Cluster 4 over Period 3 and Period 4, respectively) (Fig. 4).

The “Resurgent” group (Cluster 2) received the largest number of participants after the first month of the lockdown (Period 2)—particularly coming from Cluster 1 and 4—, increasing from 23.8 to 29.9%. Approximately, half of the participants remained stable in the same Cluster during Periods 3 (33.3%) and 4 (37.2%), while a high percentage of participants migrated to Clusters 1 and 4 during Periods 3 (24.2%) and 4 (37.2%).

The “Resilient” group (Cluster 3) showed little change during the lockdown; most of the participants remained in this cluster during the three months (41.0%, 67.9%, and 47.9% for Period 2, 3 and 4, respectively). Nevertheless, there were small migrations to other clusters (13.9%, 16.1%, and 19.7% during Periods 2, 3, and 4, respectively).

### Specific Trajectories of the Participants Within the Clusters over Time

The number of changes experienced by the participants over time were calculated considering the 51.9% of the initial sample (i.e., the 256 participants that answered the questionnaires in at least two periods). The number of changes among clusters ranged from 0 to 3: 44.9% of the participants remained in the same cluster (zero changes), 35.5% changed once, 16.0% changed twice, and 3.5% changed clusters three times.

Regarding differences in the number of changes based on sociodemographic variables, non-significant differences were found depending on: age, *F*(3, 252) = 0.30, *p* = 0.825, *η*_*p*_^2^ = 0.00; sex, χ^2^(3, *N* = 256) = 0.56, *p* = 0.894; marital status, χ^2^(15, *N* = 256) = 12.98, *p* = 0.582; diagnosis of mental illnesses, χ^2^(3, *N* = 256) = 4.84, *p* = 0.169; diagnosis of chronic illnesses, χ^2^(3, *N* = 256) = 2.79, *p* = 0.441; occupational situation, χ^2^(15, *N* = 256) = 5.54, *p* = 0.990; current employment situation, χ^2^(12, *N* = 256) = 13.08, *p* = 0.362; and if they were working as healthcare professionals, χ^2^(6, *N* = 256) = 3.80, *p* = 0.714. Significant differences in the number of changes were found depending on the monetary incomes, χ^2^(6, *N* = 256) = 12.52, *p* = 0.046. Participants that had incomes “at the mean” had a significantly greater percentage of participants (53.4%) that never changed over time (Adjusted Standardized Residuals = 2.8), while participants that had incomes “below the mean” had a significantly greater percentage of participants (45.2%) that changed once of cluster (Adjusted Standardized Residuals = 2.4). There were not significant differences in those participants that change twice or three times.

However, differences on the number of changes were found depending on the cluster in which participants started at Period 1, χ^2^(9, *N* = 256) = 18.10, *p* = 0.036. Participants initially in the “Resilient” group at Period 1 had a significantly greater percentage of participants that never changed over time (Adjusted Standardized Residuals = 3.5), while those that started in the “Thriving” group had a significantly lower percentage of participants that never changed (Adjusted Standardized Residuals = -2.8). Percentages of the trajectories that followed participants over time are shown in Table 4.

**Table 4:** Trajectories followed by the individuals over time among clusters

	N = 256^a^ (%)
“Survival” group at Period 1 (N = 56)	
Survival (no changes)	46.4
Survival → Resurgent	21.4
Survival → Resilient	8.9
Survival → Resurgent → Survival	5.4
Survival → Resilient → Survival	3.6
Survival → Resurgent → Survival → Resurgent	3.6
Survival → Thriving	1.8
Survival → Resurgent → Resilient	1.8
Survival → Thriving → Resurgent	1.8
Survival → Resurgent → Thriving	1.8
Survival → Resilient → Resurgent → Survival	1.8
Survival → Resilient → Resurgent	1.8
“Resurgent” group at Period 1 (N = 56)	
Resurgent (no changes)	35.7
Resurgent → Survival	21.4
Resurgent → Thriving	19.6
Resurgent → Thriving → Resurgent	7.1
Resurgent → Resilient	5.4
Resurgent → Resilient → Thriving	3.6
Resurgent → Thriving → Resurgent → Thriving	1.8
Resurgent → Survival → Resurgent	1.8
Resurgent → Thriving → Survival	1.8
Resurgent → Thriving → Resurgent → Survival	1.8
“Resilient” group at Period 1 (N = 93)	
Resilient (no changes)	59.1
Resilient → Thriving	12.9
Resilient → Resurgent	6.5
Resilient → Survival	6.5
Resilient → Resurgent → Thriving	4.3
Resilient → Resurgent → Resilient	2.2
Resilient → Thriving → Resilient	2.2
Resilient → Thriving → Resurgent	2.2
Resilient → Survival → Resurgent	1.1
Resilient → Thriving → Resilient → Survival	1.1
Resilient → Resurgent → Survival → Resilient	1.1
Resilient → Resurgent → Survival	1.1
“Thriving” group at Period 1 (N = 51)	
Thriving (no changes)	27.5
Thriving → Resurgent	23.5
Thriving → Resilient	21.6
Thriving → Resurgent → Thriving	7.8
Thriving → Resilient → Thriving	5.9
Thriving → Resilient → Resurgent	5.9
Thriving → Resurgent → Resilient	3.9
Thriving → Resilient → Survival → Resilient	2.0
Thriving → Resurgent → Thriving → Resurgent	2.0

^a^The shown percentages have been calculated considering the participants that answered the questionnaires in at least two periods

## Discussion

In this study, we focused on relevant positive aspects of the individual's functioning (meaning in life, gratitude, compassion, life satisfaction, resilience), as well as PTG responses and emotional distress that were susceptible to change during the first COVID-19 wave in Spain. We analyzed the evolution of different psychological variables, identified the different psychological response profiles, and assessed their temporal trajectories (or inter-cluster changes). To this end, the evolution of psychological responses of the Spanish participants was followed between March 21 and June 21, 2020, which was the period of strict confinement in our country.

Regarding the general evolution of the variables, our results confirm that an adverse and prolonged situation causes emotional distress in the general population, but the scores on depressive or anxiety symptoms did not achieve the clinical cut-off (Kroenke et al., 2007). After this initial negative response, scores improved at the end of lockdown, being even better compared to the beginning. In fact, emotional distress, even at the highest levels, did not reach clinical significance. Although the COVID-19 pandemic did not end by Period 4 (June 21), the end of the state of alarm and confinement seemed to positively affect the improvement of emotional distress. This is in line with some studies that report that emotional distress decreased during the final stages of the lockdown period (Fancourt et al., 2020); however, most studies conclude that emotional distress was high throughout the whole period (Arora et al., 2020). According to our results, anxiety was the only emotion that remained stable over time, and this emotion may be considered an adaptive mechanism in this context, given the unpredictable nature of the stressor and the need for health care during the pandemic (Xiong et al., 2020).

As for the positive functioning variables, as initially hypothesized, scores decreased from the beginning. That is, search for meaning, gratitude, resilience, and compassion decreased after the first month of confinement, with no subsequent increase. However, presence of meaning and life satisfaction were maintained over time. Dimensions of PTG showed a similar pattern, as the capacities of having more intimate relations with others, appreciating life, changing priorities, and experiencing spiritual change decreased after the first month of confinement. However, participants recognized more personal strengths at the end of the strict lockdown, supported by a significant increase of this dimension.

Regarding the profiles of psychological responses, we identified four different clusters, with similar percentages of participants by the end of the confinement: “Survival”, “Resurgent”, “Resilient”, and “Thriving”. This finding confirms that there are heterogeneous psychological responses following major life stressors (Galatzer-Levy et al., 2018). “Survivals” were the most emotionally affected by the adverse situation, with fewer positive resources and no personal improvement. “Resurgent” individuals had similar emotional responses to the that of “Survivals”, but with more positive psychological resources and greater “growth” after this situation, as they were the second cluster experiencing the highest PTG. “Resilients” showed low growth, although this situation did never have a significant negative impact and they always had positive resources. Finally, the “Thriving” showed some emotional discomfort, had positive resources, “learned” to the greatest extent from this situation, and achieved some personal transformation. As regards to the differences between clusters depending on age and sex, we found that, overall, the “Resilient” group was the cluster that gathered the oldest individuals (i.e., mean age of around 40–45 years) and men over periods (i.e., around 50–60% of men).

The “Resilient” group was the cluster that gathered the largest number of individuals from the beginning to the end of the study period (30–37% of the participants). That is, one third of our sample scored high in positive functioning variables and did not experience significant emotional distress. This result corroborates that, despite the adverse nature of the pandemic, most individuals are likely to be resilient or to have a stable trajectory of mental health (Chen & Bonanno, 2020; Galatzer-Levy et al., 2018). As for PTG, this group did not show any relevant change, and this finding may be related to the high initial scores in positive functioning. In fact, the lower scores for PTG were for the “Resilient” group, while the higher scores were obtained for the “Thriving” group. It is worth noting that the “Resilient” group also scored higher on positive affect and lower on emotional distress than the “Thriving” group. This may open up the debate on the need of experiencing a combination of moderate emotional distress and positive affect in order to experience PTG; mixed emotions are commonly reported in response to negative events (Hui et al., 2009; Scott et al., 2014). Moreover, positive emotions constitute an active ingredient in resilient individuals that may help them flourish despite their struggle with a crisis (Fredrickson et al., 2003). Overall, our results do not necessarily imply that the “Resilient” group did not achieve a positive transformation following the situation; however, our findings suggest that the growth is higher when the emotional impact is greater at the beginning, as seen for the “Thriving” group.

Other variables that differentiate the “Resilient” and “Thriving” groups are the higher levels of resilience and search for meaning experienced by the “Thriving” group over time. A possible explanation for this is that the combination of higher resilience and the search for meaning in life enhance the experience of PTG. In previous studies, the search for meaning has been associated with well-being among individuals who already had an important meaning in their life (Park et al., 2010). In this regard, the experience of PTG seems to be more likely to occur in individuals that search for further meaning and with robust positive functioning variables.

Regarding the differences between the “Thriving” and “Resurgent” groups, it should be noted that the scoring in “Resurgent” individuals was higher for emotional distress and lower on PTG, but also lower on positive functioning variables. As for the “Survival” group, they showed an emotional distress that was similar to that of the “Resurgent” group, and a low PTG similar to the “Resilient” group. Moreover, they had the lowest scores in positive functioning variables.

Overall, cluster results point out that higher emotional distress was associated with lower scores in positive functioning variables. The scores of “Resilient” and “Thriving” individuals were higher on positive functioning variables and lower on emotional distress. Both profiles are in line with studies that suggest that meaning of life and life satisfaction may be buffers against the negative effects of a threat as unpredictable as the pandemic (Lin, 2020; Trzebiński et al., 2020). Along the same lines, other studies show that individuals with higher levels of resilience are less affected by the exposure to stress at the initial stage of the pandemic, due to better psychological functioning (Havnen et al., 2020; Kavčič et al., 2020; Lenzo et al., 2020). On the contrary, the “Survival” and the “Resurgent” groups showed lower levels of positive functioning variables and higher levels of emotional distress. Thus, the resources everyone has may act as protective factors when facing adversity (Grych et al., 2015).

Regarding the trajectories, our findings show that psychological responses remained relatively stable throughout the three months of confinement, as the analyses indicated that most individuals remained in the same cluster over time (i.e., 44.9% remained in the same cluster and 35.5% only changed once of cluster). More specifically, 59.1% of the “Resilient” group, 46.4% of the “Survival” group, 35.7% of the “Resurgent” group, and 27.5% of the “Thriving” group remained in the initial cluster. There were no significant differences in the number of changes among clusters according to any sociodemographic variable, except for income levels (i.e., individuals with income levels “at the mean” had a significantly greater percentage of participants that never changed over time, while participants that had incomes “below the mean” had a significantly greater percentage of participants that changed once of cluster). Nevertheless, some migrations occurred after the first month of confinement (e.g., the number of individuals increased in the “Resurgent” group). Furthermore, an important migration occurred again from the second month of confinement to the end of the state of alarm; the profile of initially “Resurgent” individuals changed to a “Survival” or “Thriving” profile. By the end of the study period, the percentage of individuals in the four clusters was similar, although slightly higher in the “Resilient” group. Thus, regarding the most common trajectories of the participants (i.e., > 10.0%), the findings showed that: (1) individuals that started in the “Survival” group changed to “Resurgent” (21.4%); (2) individuals that started in the “Resurgent” group changed to “Survival” (21.4%) or “Thriving” (19.6%); (3) individuals that started as “Resilient” changed to “Thriving” (12.9%), and (4) the individuals that started in “Thriving'' changed to “Resurgent” (23.5%) or “Resilient” (21.6%). It should be noted that a “graded” progression between the migrations appears to exist, passing “step by step” from one cluster to another, so if individuals were characterized by being a “Survival” they did not change to “Thriving”, and the same happened with initially “Thriving” and “Resilient” individuals, which did not become “Survivals”. However, “Resurgent” individuals may improve their profile (“Thriving”) or worsen it (“Survival”). It would be very valuable to learn more about what triggers these individuals to shift to one pathway or the other.

The main strength of this study is the exploration made to outline the different responses to the enforced and mandatory confinement and subsequent de-escalation, considering positive and negative variables. We were able to identify how positive functioning and emotional distress changed over time and how these factors related to PTG. Nevertheless, there are limitations to this study. The first one is related to the representativeness of the sample; participants were volunteers recruited through social media and the percentage of female/male and age-ranges are not balanced. The second limitation is the high attrition rate; only 35% of the participants completed all measurements at the end of the study. Thirdly, these findings should be interpreted with caution, as the clusters have been determined using a statistical criterion, not a theoretical one. Infurna and Luthar (2018) concluded that the statistical decisions and measurement choices have an important impact on the conclusions in the field of resilience (e.g., when using Growth Mixture Modeling, the assumptions made can influence the number of identified trajectories or the proportion of individuals in each category), so the authors pointed out the need to replicate the findings across samples and measures. Finally, it should be noted that the lack of measurements beyond the three months of the confinement restricts the conclusions of the different psychological trajectories, as it is possible that the “delayed onset” trajectory arose later among some participants. Given the long duration of the COVID-19 pandemic, future studies should analyze the prevalence of the delayed onset trajectory (i.e., elevation in emotional distress that emerges following a significant delay), which has been found in previous studies (Galatzer-Levy et al., 2018).

Preliminary clinical implications may be drawn from this study. Our data suggest that there are different ways (at least four) to respond to a (mandatory) confinement and that the observed initial responses are relatively stable over time. The passing of time does not seem to be enough to overcome the stressor, particularly in individuals who are struggling the most. In this sense, early detection of profiles and tailored interventions aimed at reducing the risk of emotional distress, but also increasing positive functioning variables and promoting PTG can be crucial to prevent the effects of a prolonged stressor or even experiencing a transformation. More specifically, the “Survival” group could be a target population of preventive interventions to provide psychological resources for reducing emotional distress and increasing positive functioning variables. Moreover, the “Resurgent” group could be the population target by preventive interventions for promoting PTG, as going a step further in “growing” may help them face future stressful conditions with lower emotional distress and higher positive functioning variables. Finally, the “Resilient” and “Thriving” groups could be targeted for participating in interventions aimed at reinforcing their positive functioning variables (especially, gratitude, resilience and search for meaning).

In sum, analyzing the different variables jointly may allow us to better understand the responses to a prolonged and adverse situation in terms of positive functioning variables, emotional distress, and PTG. In this regard, lower emotional distress seems to arise in combination with higher positive functioning variables (e.g., gratitude and resilience), while higher PTG seems to appear when moderate emotional distress and positive affect are combined, as well as higher positive functioning variables, such as resilience and search for meaning. Hence, this study gives a wider perspective of both negative and positive psychological reactions to a tremendous stressor experienced worldwide.

## Supplementary Information

Below is the link to the electronic supplementary material.

Supplementary file1 (PNG 12 KB)

Supplementary file2 (PNG 20 KB)

Supplementary file3 (PNG 277 KB)

bv

## Data Availability

Raw data is available at the Open Science Framework https://osf.io/9f3wg/.

## Footnotes

1 For the assessment, we chose four key points related to the changes in the confinement measures imposed by the Spanish government that affected the daily lives of people. The four key points were: (1) when the strictest measures were implemented; (2) when the strictest measures ended, but confinement was still mandatory; (3) when the de-escalation started (i.e., outside activities started to be progressively allowed); and (4) when the “new normality” started (i.e., the confinement ended, but some prevention measures were still mandatory; e.g., wearing a mask).

2 The number of clusters were not established a priori, as given the unprecedented stressor of the COVID-19 pandemic, the clusters will be constructed using an exploratory analysis relying on a statistical criterion.

3 Only participants who completed all the questionnaires for each study period were considered for the analyses.

4 According to Eisinga et al. (2013), we calculated Spearman-Brown for the two-item scales, as it is less biased -particularly if the correlation is relatively strong- than Cronbach’s alpha.

5 N of Period 1 is equal to the number of participants who completed all the measurements in Period 1; N of Period 2 is equal to the number of participants who completed all the measurements in Period 1 and Period 2; N of Period 3 is equal to the number of participants who completed all the measures in Period 1 and Period 3; and N of time 4 is equal to the number of participants who completed all the measurements in Period 1 and Period 4.

6 Given that linear mixed models use the full data set, the sample size for Period 1 is N = 459 in all the variables, but for Periods 2–4 there are slight differences in the N in comparison to the values reported in Table 2 and in the subsequent clustering analyses.

## References

[CR1] Arora T, Grey I, Östlundh L, Lam KBH, Omar OM, Arnone D (2020). The prevalence of psychological consequences of COVID-19: A systematic review and meta-analysis of observational studies. Journal of Health Psychology.

[CR2] Bonanno GA (2004). Loss, trauma, and human resilience: Have we underestimated the human capacity to thrive after extremely aversive events?. American Psychologist.

[CR3] Bonanno GA, Galea S, Bucciarelli A, Vlahov D (2006). Psychological resilience after disaster: New York City in the aftermath of the September 11th terrorist attack. Psychological Science.

[CR4] Bonanno GA, Ho SM, Chan JC, Kwong RS, Cheung CK, Wong CP, Wong VC (2008). Psychological resilience and dysfunction among hospitalized survivors of the SARS epidemic in Hong Kong: A latent class approach. Health Psychology.

[CR5] Bonanno GA, Mancini AD (2012). Beyond resilience and PTSD: Mapping the heterogeneity of responses to potential trauma. Psychological Trauma: Theory, Research, Practice, and Policy.

[CR6] Bonanno GA, Rennicke C, Dekel S (2005). Self-enhancement among high-exposure survivors of the September 11th terrorist attack: Resilience or social maladjustment?. Journal of Personality and Social Psychology.

[CR7] Campbell-Sills L, Stein MB (2007). Psychometric analysis and refinement of the connor–davidson resilience scale (CD-RISC): Validation of a 10-item measure of resilience. Journal of Traumatic Stress.

[CR8] Cann A, Calhoun LG, Tedeschi RG, Taku K, Vishnevsky T, Triplett KN, Danhauer SC (2010). A short form of the Post-traumatic Growth Inventory. Anxiety Stress and Coping.

[CR9] Cárdenas M, Barrientos J, Ricci E, Páez D (2015). Spanish adaptation and validation of the post-traumatic growth inventory–short form. Violence and Victims.

[CR10] Chen S, Bonanno GA (2020). Psychological adjustment during the global outbreak of COVID-19: A resilience perspective. Psychological Trauma: Theory, Research, Practice, and Policy.

[CR11] Chiesi F, Lau C, Saklofske DH (2020). A revised short version of the compassionate love scale for humanity (CLS-H-SF): Evidence from item response theory analyses and validity testing. BMC Psychology.

[CR12] Clatworthy J, Buick D, Hankins M, Weinman J, Horne R (2005). The use and reporting of cluster analysis in health psychology: A review. British Journal of Health Psychology.

[CR13] Diener E, Emmons RA, Larsen RJ, Griffin S (1985). The satisfaction with life scale. Journal of Personality Assessment.

[CR14] Duan L, Zhu G (2020). Psychological interventions for people affected by the COVID-19 epidemic. The Lancet Psychiatry.

[CR15] Eisinga R, Grotenhuis MT, Pelzer B (2013). The reliability of a two-item scale: Pearson, Cronbach, or Spearman-Brown?. International Journal of Public Health.

[CR16] Fancourt D, Steptoe A, Bu F (2020). Trajectories of anxiety and depressive symptoms during enforced isolation due to COVID-19 in England: A longitudinal observational study. The Lancet Psychiatry.

[CR17] Forgy EW (1965). Cluster analysis of multivariate data: Efficiency vs interpretability of classifications. Biometrics.

[CR18] Fredrickson BL, Tugade MM, Waugh CE, Larkin GR (2003). What good are positive emotions in crisis? A prospective study of resilience and emotions following the terrorist attacks on the United States on September 11, 2001. Journal of Personality and Social Psychology.

[CR19] Galatzer-Levy IR, Huang SH, Bonanno GA (2018). Trajectories of resilience and dysfunction following potential trauma: A review and statistical evaluation. Clinical Psychology Review.

[CR20] Galea, S., Vlahov, D., Resnick, H., Ahern, J., Susser, E., Gold, J., Bucuvalas, M., & Kilpatrick, D. (2003). Trends of probable post-traumatic stress disorder in New York City after the September 11 terrorist attacks. *American Journal of Epidemiology, 158*(6), 514–524. 10.1093/aje/kwg187. 10.1093/aje/kwg187 12965877

[CR21] García-Campayo J, Zamorano E, Ruiz MA, Pérez-Páramo M, López-Gómez V, Rejas J (2012). The assessment of generalized anxiety disorder: Psychometric validation of the Spanish version of the self-administered GAD-2 scale in daily medical practice. Health and Quality of Life Outcomes.

[CR22] Grant RW, McCloskey J, Hatfield M, Uratsu C, Ralston JD, Bayliss E, Kennedy CJ (2020). Use of latent class analysis and k-Means clustering to identify complex patient profiles. JAMA Network Open.

[CR23] Grych J, Hamby S, Banyard V (2015). The resilience portfolio model: Understanding healthy adaptation in victims of violence. Psychology of Violence.

[CR24] Hartigan, J., & Wong, M. (1979). Algorithm AS 136: A K-Means clustering algorithm. *Journal of the Royal Statistical Society. Series C (Applied Statistics), 28*(1), 100–108. 10.2307/2346830.

[CR25] Hastie T, Tibshirani R, Friedman J (2017). The elements of statistical learning: Data mining, inference, and prediction.

[CR26] Havnen A, Anyan F, Hjemdal O, Solem S, Gurigard Riksfjord M, Hagen K (2020). Resilience moderates negative outcome from stress during the COVID-19 pandemic: A moderated-mediation approach. International Journal of Environmental Research and Public Health.

[CR27] Ho S. M. Y. (2016) Post-Traumatic growth: Focus on concepts and cross-cultural measurement issues. In C. Martin, V. Preedy & V. Patel (Eds.), *Comprehensive Guide to Post-Traumatic Stress Disorders* (pp. 1831–1848). Springer. 10.1007/978-3-319-08359-9_60.

[CR28] Hobfoll SE, Palmieri PA, Johnson RJ, Canetti-Nisim D, Hall BJ, Galea S (2009). Trajectories of resilience, resistance, and distress during ongoing terrorism: The case of Jews and Arabs in Israel. Journal of Consulting and Clinical Psychology.

[CR29] Hui CM, Fok HK, Bond MH (2009). Who feels more ambivalence? Linking dialectical thinking to mixed emotions. Personality and Individual Differences.

[CR65] Infurna, F. J., & Luthar, S. S. (2018). Re-evaluating the notion that resilience is commonplace: A review and distillation of directions for future research, practice, and policy. *Clinical Psychology Review,**65*, 43–56. 10.1016/j.cpr.2018.07.003 10.1016/j.cpr.2018.07.003 30125756

[CR30] Kavčič T, Avsec A, Kocjan GZ (2020). Psychological functioning of Slovene adults during the COVID-19 Pandemic: Does resilience matter?. Psychiatric Quarterly.

[CR31] Kroenke K, Spitzer RL, Williams JB (2003). The Patient Health Questionnaire-2: Validity of a two-item depression screener. Medical Care.

[CR32] Kroenke K, Spitzer RL, Williams JB, Monahan PO, Löwe B (2007). Anxiety disorders in primary care: Prevalence, impairment, comorbidity, and detection. Annals of Internal Medicine.

[CR33] Lau JT, Yang X, Tsui HY, Pang E, Wing YK (2006). Positive mental health-related impacts of the SARS epidemic on the general public in Hong Kong and their associations with other negative impacts. Journal of Infection.

[CR34] Lenzo V, Quattropani MC, Musetti A, Zenesini C, Freda MF, Lemmo D, Vegni E, Borghi L, Plazzi G, Castelnuovo G, Cattivelli R, Saita E, Franceschini C (2020). Resilience contributes to low emotional impact of the COVID-19 outbreak among the general population in Italy. Frontiers in Psychology.

[CR35] Lepore SJ, Revenson TA, Calhoun LG, Tedeschi RG (2006). Resilience and post-traumatic growth: Recovery, resistance, and reconfiguration. Handbook of post-traumatic growth: Research and practice.

[CR36] Lin L (2020). Longitudinal associations of meaning in life and psychosocial adjustment to the COVID-19 outbreak in China. British Journal of Health Psychology.

[CR37] López-Gómez I, Hervás G, Vázquez C (2015). An adaptation of the Positive and Negative Affect Schedules (PANAS) in a Spanish general sample. Behavioral Psychology.

[CR38] Magallares A, Recio P, Sanjuán P (2018). Factor structure of the Gratitude Questionnaire in a Spanish Sample. The Spanish Journal of Psychology.

[CR39] Malhotra M, Chebiyan S (2016). Posttraumatic growth: Positive changes following adversity-an overview. International Journal of Psychology and Behavioral Sciences.

[CR40] McCleary J, Figley C (2017). Resilience and trauma: Expanding definitions, uses, and contexts. Traumatology.

[CR41] McCullough, M. E., Emmons, R. A., & Tsang, J. A. (2002). The grateful disposition: A conceptual and empirical topography. *Journal of Personality and Social Psychology, 82*, 112–127. 10.1037/0022-3514.82.1.112. 10.1037//0022-3514.82.1.112 11811629

[CR42] Neria, Y., Nandi, A., & Galea, S. (2008). Post-traumatic stress disorder following disasters: A systematic review. *Psychological Medicine, 38*(4), 467–480. 10.1017/S0033291707001353 10.1017/S0033291707001353 PMC4877688 17803838

[CR43] Notario-Pacheco B, Solera-Martínez M, Serrano-Parra MD, Bartolomé-Gutiérrez R, García-Campayo J, Martínez-Vizcaíno V (2011). Reliability and validity of the Spanish version of the 10-item Connor-Davidson Resilience Scale (10-item CD-RISC) in young adults. Health and Quality of Life Outcomes.

[CR44] Odriozola-González P, Planchuelo-Gómez Á, Irurtia MJ, de Luis-García R (2020). Psychological symptoms of the outbreak of the COVID-19 confinement in Spain. Journal of Health Psychology.

[CR45] Park N, Park M, Peterson C (2010). When is the search for meaning related to life satisfaction?. Applied Psychology: Health and Well-Being.

[CR46] Rodríguez-Muñoz, M. D. L. F., Castelao, P. L., Olivares, M. C., Soto, C. B., Izquierdo, N. M., Ferrer, F. B., & Huynh-Nhu, L. (2017). PHQ-2 as first screening instrument of prenatal depression in primary health care, Spain. *Revista Española de Salud Pública, 91*, e201701010.28134236

[CR47] Roy D, Tripathy S, Kar SK, Sharma N, Verma SK, Kaushal V (2020). Study of knowledge, attitude, anxiety & perceived mental healthcare need in Indian population during COVID-19 pandemic. Asian Journal of Psychiatry.

[CR48] Scott SB, Sliwinski MJ, Mogle JA, Almeida DM (2014). Age, stress, and emotional complexity: Results from two studies of daily experiences. Psychology and Aging.

[CR49] Smith KV, Ehlers A (2020). Cognitive predictors of grief trajectories in the first months of loss: A latent growth mixture model. Journal of Consulting and Clinical Psychology.

[CR50] Steger MF, Frazier P, Oishi S, Kaler M (2006). The Meaning in Life Questionnaire: Assessing the presence of and search for meaning in life. Journal of Counseling Psychology.

[CR51] Steger MF, Frazier PA, Zacchanini JL (2008). Terrorism in two cultures: Stress and growth following September 11 and the Madrid train bombings. Journal of Loss and Trauma.

[CR52] Tamiolaki A, Kalaitzaki AE (2020). “That which does not kill us, makes us stronger”: COVID-19 and Post-traumatic Growth. Psychiatry Research.

[CR53] Tedeschi RG, Calhoun LG (2004). Target article: “Posttraumatic growth: Conceptual foundations and empirical evidence”. Psychological Inquiry.

[CR54] Torales J, O’Higgins M, Castaldelli-Maia JM, Ventriglio A (2020). The outbreak of COVID-19 coronavirus and its impact on global mental health. International Journal of Social Psychiatry.

[CR55] Trzebiński J, Cabański M, Czarnecka JZ (2020). Reaction to the COVID-19 pandemic: The influence of meaning in life, life satisfaction, and assumptions on world orderliness and positivity. Journal of Loss and Trauma.

[CR56] Tugade MM, Fredrickson BL (2004). Resilient individuals use positive emotions to bounce back from negative emotional experiences. Journal of Personality and Social Psychology.

[CR57] Updegraff JA, Silver RC, Holman EA (2008). Searching for and finding meaning in collective trauma: Results from a national longitudinal study of the 9/11 terrorist attacks. Journal of Personality and Social Psychology.

[CR58] Vázquez, C., Duque, A., & Hervás, G. (2013). Satisfaction with Life Scale in a representative sample of Spanish adults: validation and normative data. *The Spanish Journal of Psychology, 16*, 1–15. 10.1017/sjp.2013.82. 10.1017/sjp.2013.82 24230945

[CR59] Vázquez C, Hervás G (2010). Terrorist attacks and benefit finding: The role of positive and negative emotions. Journal of Positive Psychology.

[CR60] Vernon LL, Dillon JM, Steiner AR (2009). Proactive coping, gratitude, and posttraumatic stress disorder in college women. Anxiety, Stress, and Coping.

[CR61] Watson D, Clark LA, Tellegen A (1988). Development and validation of brief measures of positive and negative affect: The PANAS Scales. Journal of Personality and Social Psychology.

[CR62] World Medical Association (2013). World Medical Association Declaration of Helsinki: Ethical principles for medical research involving human subjects. JAMA.

[CR63] Xiong J, Lipsitz O, Nasri F, Lui LM, Gill H, Phan L, Chen-Li D, Iacobucci M, Ho R, Majeed A, McIntyre RS (2020). Impact of COVID-19 pandemic on mental health in the general population: A systematic review. Journal of Affective Disorders.

[CR64] Zakharov, K. (2016). Application of k-means clustering in psychological studies. *Tutorials in Quantitative Methods for Psychology*, *12*(2), 87–100. 10.20982/tqmp.12.2.p087

