# Supplementary figures and images for: Exploring the Heterogeneity and Trajectories of Positive Functioning Variables, Emotional Distress, and Post-traumatic Growth During Strict Confinement Due to COVID-19
Source: J Happiness Stud. 2021 Nov 2;23(4):1683–708. doi: 10.1007/s10902-021-00469-z (PMC8561082; doi:10.1007/s10902-021-00469-z)

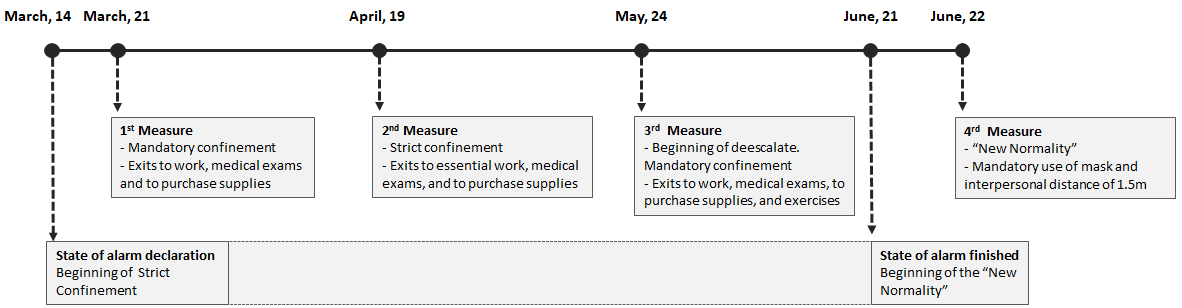

Supplement: Supplementary file 1 — Supplementary file1 (PNG 12 KB) [file 10902_2021_469_MOESM1_ESM.png]

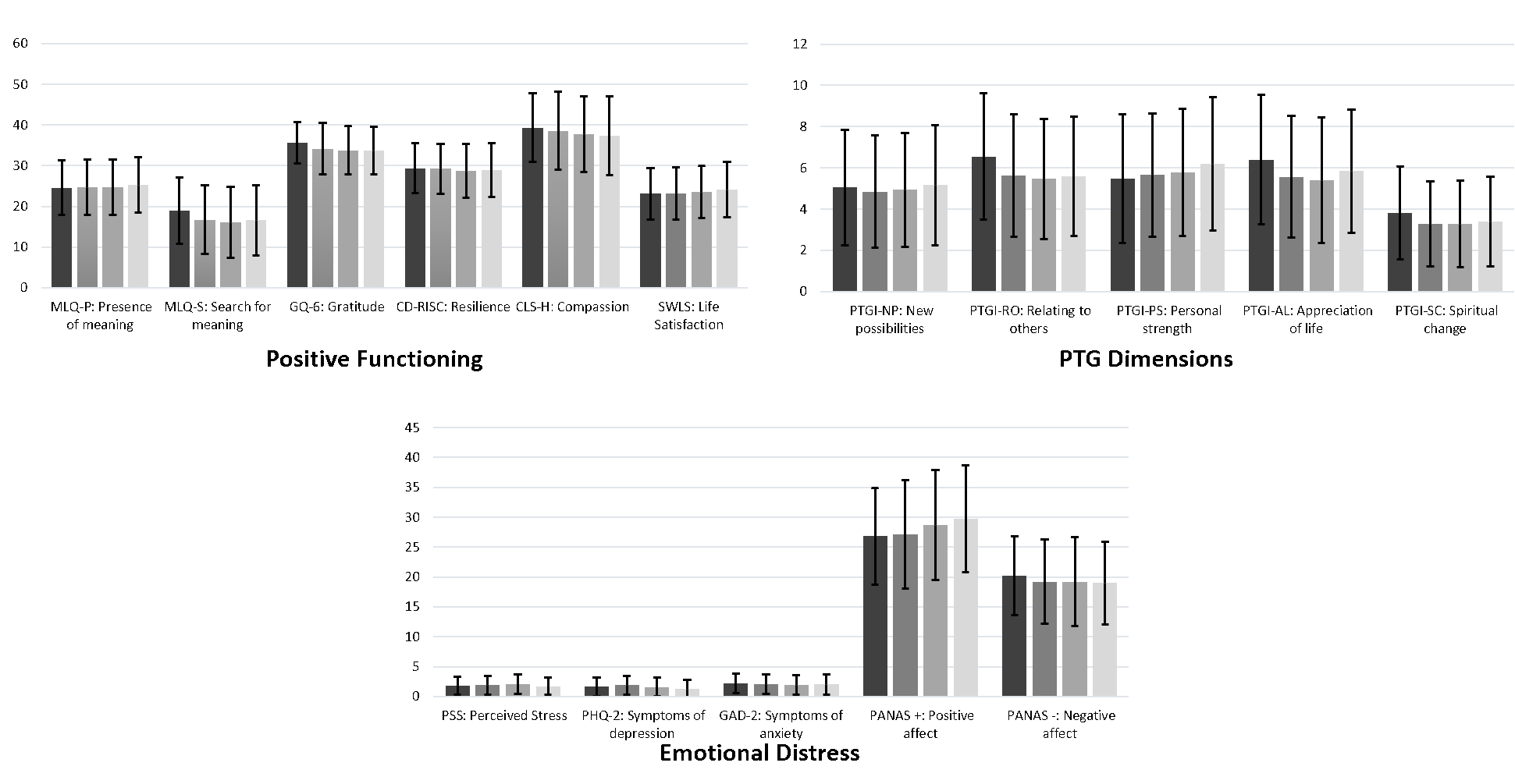

Supplement: Supplementary file 2 — Supplementary file2 (PNG 20 KB) [file 10902_2021_469_MOESM2_ESM.png]

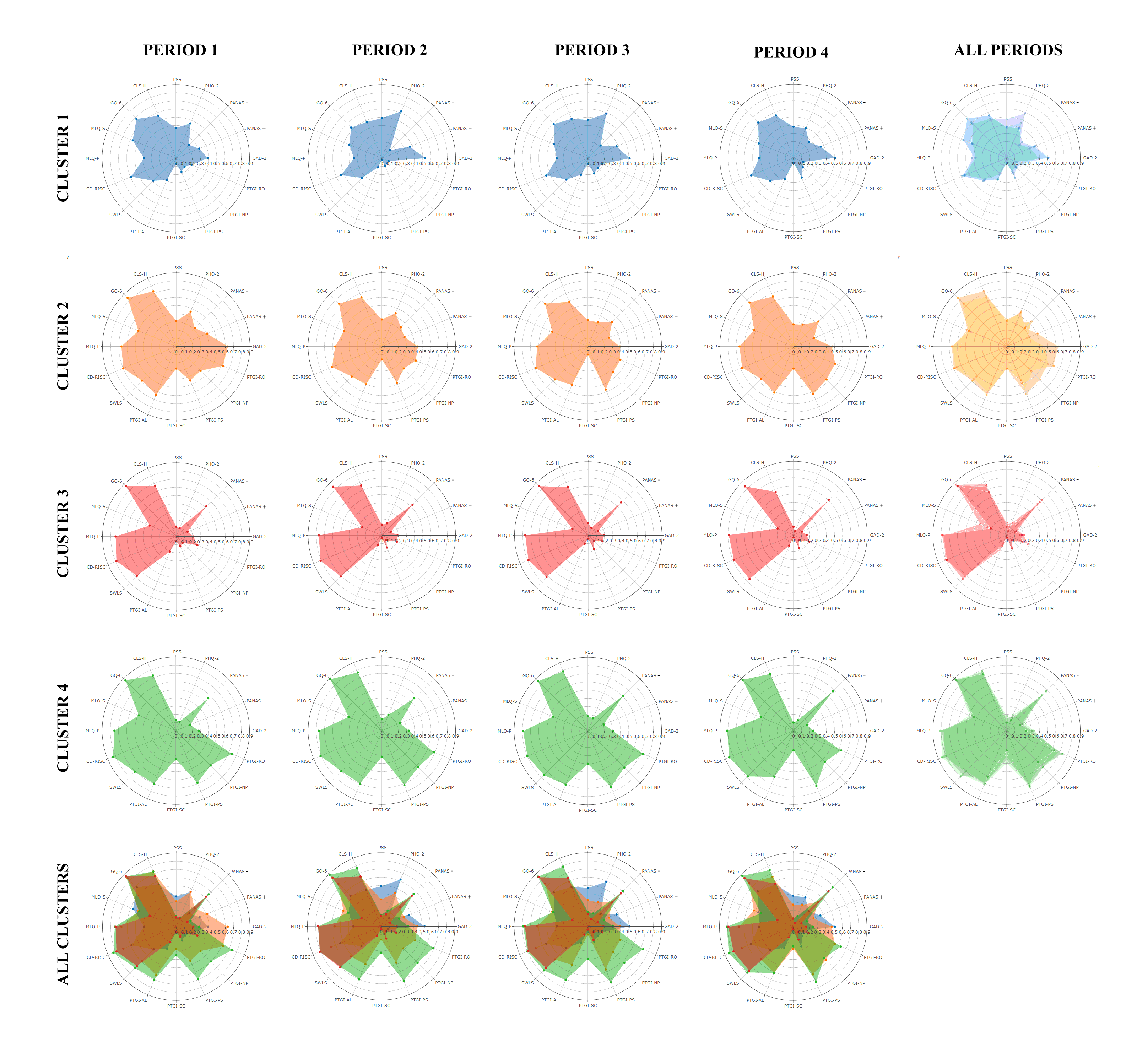

Supplement: Supplementary file 3 — Supplementary file3 (PNG 277 KB) [file 10902_2021_469_MOESM3_ESM.png]
